# Evaluating the effectiveness of a focused CBT training for panic disorder: a randomized parallel trial

**DOI:** 10.1017/S0033291725102353

**Published:** 2025-11-24

**Authors:** Saarim Yasin Aslam, Angie Jenkin, Tiago Zortea, Charlie Wykes, Samantha Sadler, Paul M. Salkovskis

**Affiliations:** 1 Department of Experimental Psychology, University of Oxford, Oxford, UK; 2 Central and North West London Mental Health NHS Trust, London, UK; 3 Oxford Health NHS Foundation Trust, Oxford, UK

**Keywords:** catastrophic misinterpretations, CBT, panic disorder, safety-seeking behaviors

## Abstract

**Background:**

Recovery rates for panic disorder in NHS Talking Therapies (NHSTT) services in the United Kingdom do not match those in randomized trials. Previous research has found that training therapists in ‘focused cognitive behavioral therapy’ (CBT) improves outcomes. The primary aim was to examine whether focused CBT delivered by trained psychological well-being practitioners (PWPs) can improve treatment outcomes for panic disorder. An exploratory aim was to evaluate the potential impact of a novel component of focused CBT, which includes the use of ‘approach-supporting behaviors’ (ASBs) where safety-seeking behaviors (SSBs) are prominent.

**Methods:**

We conducted a randomized parallel trial. Participants were randomly allocated to focused CBT or the current treatment at ‘Step Two’ (treatment as usual) in two NHSTT services (ISRCTN:11268881).

**Results:**

We found a significant group-timepoint interaction. Those in focused CBT had significantly greater reductions in the primary measure of panic severity relative to those in treatment as usual (TAU). The level of ASBs did not predict a change in panic severity; however, the level of SSBs at the end of treatment did predict a change in panic severity.

**Conclusions:**

Focused CBT is effective for panic disorder and is superior to TAU, supporting the applicability of this lower-intensity and panic-specific version of CBT for panic disorder.

## Introduction

Panic disorder is a common problem and is defined as recurrent panic attacks, at least some of which occur unexpectedly in the absence of particular stimuli, and individuals may fear the recurrence of panic attacks and their consequences (American Psychiatric Association, 2022). Worldwide prevalence rates for panic disorder are reported to be around 1.7% (De Jonge et al., 2016; Skapinakis et al., 2011). Panic disorder can be particularly disabling, along with agoraphobia, a marked fear of places where help may not be available and/or from which escape might be difficult (APA, 2022). In agoraphobia, anxiety around these situations occurs due to a fear of negative outcomes when in these situations, such as panic attacks, hence panic disorder and agoraphobia can commonly occur together (APA, 2022).

The cognitive theory of panic disorder states that ‘panic attacks result from the catastrophic misinterpretation of certain bodily sensations. The sensations which are misinterpreted are mainly those which are involved in normal anxiety responses (e.g. palpitations, breathlessness, dizziness, etc.), but also include some other bodily sensations. The catastrophic misinterpretation involves perceiving these sensations as much more dangerous than they really are’ (Clark, 1986, p.462). For example, perceiving an increase in heart rate (bodily sensation) as indicating a heart attack (perceived catastrophe) (Clark & Ehlers, 1993). Individuals with panic disorder have a relatively enduring tendency to catastrophically misinterpret normal bodily sensations as an immediate, impending mental or physical disaster (Clark & Ehlers, 1993).

Panic disorder may also be maintained by safety-seeking behaviors (SSBs) in which people engage with behaviors to try to avoid, escape, or prevent a perceived catastrophe (Salkovskis, 1991; Salkovskis, Clark, & Gelder, 1996). For example, individuals with panic disorder might keep still or ask for help to prevent the feared catastrophe of a heart attack (Salkovskis et al., 1996). However, this prevents individuals from obtaining disconfirmatory evidence and potentially (but not necessarily) provides immediate anxiety relief. This signals that one must engage with their SSBs to stop the feared catastrophe (Salkovskis, Clark, Hackmann, Wells, & Gelder, 1999). Therefore, catastrophic misinterpretations encourage engagement with SSBs, preventing recovery (Salkovskis, Hackmann, Wells, Gelder, & Clark, 2007).

For psychological treatment to be effective for panic disorder, understanding the role of catastrophic misinterpretations and SSBs is crucial (Salkovskis et al., 2007). Treatment should help generate convincing disconfirmations of one’s catastrophic misinterpretations to help reduce panic symptoms by dropping SSBs. Research supports this approach; individuals who received cognitive behavioral therapy (CBT) targeting belief disconfirmation and SSB reduction exhibited greater improvements in anxiety, panic, and avoidance (Clark et al., 1994, 1999; Salkovskis et al., 1999, 2007). CBT targeting belief disconfirmation and SSB reduction was also more effective compared to individuals undergoing behavioral exposure alone (Salkovskis et al., 2007). A recent systematic narrative review of the cognitive theory of panic disorder found that when belief in catastrophic misinterpretations and using SSBs are reduced during treatment, this results in a significant decrease in panic symptoms and anxiety (Aslam, Zortea, & Salkovskis, 2024).

Due to its strong evidence base, the National Institute for Health and Care Excellence (NICE) recommends that treatment for panic disorder is based on CBT principles (NICE, 2020), and CBT is used in NHS Talking Therapies (NHSTT) services (formally Improving Access to Psychological Therapies (IAPT)) in England. At ‘Step Two’ care in NHSTT, which is for ‘mild to moderate’ panic disorder, this involves individual guided self-help based on CBT principles or computerized CBT (cCBT). If an individual does not improve with Step Two treatment, or if their panic disorder presentation is severe, they may receive ‘Step Three’ intervention (1:1 CBT).

Current recovery rates for panic disorder in NHSTT are substantially below RCT findings of 70–90% recovery rates 6 months post-treatment (Barlow, Gorman, Shear, & Woods, 2000; Clark et al., 1999). The national average recovery rate for panic disorder at Step Two care in NHSTT England is 43%. At the time of planning the study, in two NHSTT services within this study, the recovery rate for panic disorder was 45% and 49.5% at Step Two. This improved to 54% and 52% within the last year based on either panic-specific or general measures. Previous research has found that delivering brief focused cognitive therapy effectively reduces panic symptoms, anxiety, and depression, with no difference observed between brief vs full cognitive therapy (Clark et al., 1999). Brief cognitive therapy involved five sessions (using self-help modules before each session) over 3 months, compared to 12 weekly sessions, and CBT components were first introduced using self-help workbooks and written exercises (Clark et al., 1999). Other research found that therapists working in primary care can be trained in a way that improves outcomes in panic disorder, including panic severity and anxiety (Grey, Salkovskis, Quigley, Clark, & Ehlers, 2008).

The cognitive-behavioral treatment for panic, when properly delivered, achieves success rates of 70–90% (Clark et al., 1999). Other than shortening treatment, there have been few further developments. However, there has been significant theoretical clarification of issues surrounding SSBs, indicating that treatment may be facilitated by the judicious use of ‘approach-supporting behaviors’ (ASBs). These are behaviors which support people to approach the feared stimulus/situation, not prevent a catastrophe (Salkovskis & Millar, 2016). Therefore, the specific intention behind the behavior determines whether it is safety-seeking or not (Salkovskis & Millar, 2016; Thwaites & Freeston, 2005). Nonetheless, research has not investigated the role of ASBs vs SSBs in the treatment of panic disorder.

### Aims and hypotheses

The primary aim was to evaluate the feasibility and impact of providing training on a focused CBT intervention for panic disorder at Step Two, with the aim of improving outcomes. We hypothesized:

1. Individuals with panic disorder with or without agoraphobia who receive focused CBT treatment delivered by trained Psychological Wellbeing Practitioners (PWPs) will show greater reduction in panic symptom severity compared to those who receive the current Step Two psychological treatment.
2. Individuals with panic disorder with or without agoraphobia who receive focused CBT treatment from trained PWPs will show greater reduction in anxiety and depression, and improved daily functioning, compared to individuals receiving current Step Two psychological treatment.
3. In addition, as there is no current research exploring the link between ASBs and recovery rates in panic disorder, an exploratory aim was to monitor potential mechanisms underlying this change (SSBs and ASBs).

## Method

### Design

We conducted a randomized parallel trial, comparing the current Step Two psychological treatment for panic disorder in two NHSTT services in England, with a treatment based on Clark et al. (1999) methodology, known as ‘Focused CBT’. Ethical approval was obtained by the Cornwall & Plymouth Research Ethics Committee (23/SW/0151). The trial was pre-registered with the ISRCTN (11268881).

### Participants

#### Trial participants

Participants were those with panic disorder referred and accepted for psychological treatment at two NHSTT services. Inclusion criteria were as follows: (1) age 18+, (2) English speaking and able to complete questionnaires and workbooks in English, (3) any gender, (4) the presence of recurrent panic attacks whereby some are unexpected, and (5) panic disorder with or without agoraphobia is the main problem. Exclusion criteria were: (1) panic was not the primary difficulty, (2) the individual did not have capacity to consent, (3) the individual had a long-term physical health condition, (4) those who were involved in another research project, (5) risk/safeguarding could not be managed, (6) substance/alcohol use that would impact on therapy and the individual was unwilling to work to reduce this use and (7) inability to access materials, for example, technology barriers.

In the trial, as participants were seen at Step Two (low intensity) in NHSTT, specific agoraphobic data were not available, as those with clearly identified agoraphobia are seen at Step Three (higher intensity). Therefore, within this framework, panic disorder with or without agoraphobia is not routinely differentiated at assessment, at the specification of problem descriptors, or throughout Step Two treatment, and these service guidelines and procedures were adhered to. We therefore did not separate panic with agoraphobia from panic disorder alone.

#### Trial therapists

PWPs delivered the interventions as this was standard procedure for Step Two treatment for panic disorder; in the services involved, the majority of panic patients are treated by PWPs. PWPs were qualified, mixed in experience, and prior to randomization were identified by clinical leads of the NHSTT services following PWPs submitting an expression of interest form. Informed consent for their participation was obtained via an online consent form on Microsoft Forms.

### Randomization

Therapists (PWPs) were randomized to treatment conditions using a random number generator. The experience of PWPs was not known by the researchers prior to randomization to avoid bias.

Panic participants (service users) were randomly assigned in a 1:1 allocation ratio to either focused CBT or ‘treatment as usual’ (TAU), which included either cCBT or Guided Self Help (GSH). The randomization sequence was generated through an online system called ‘Sealed Envelope’ by a member of the research team, and randomization was completed in blocks of four, stratified by site. Participants were enrolled by research assistants and the chief investigator. Allocation concealment was achieved through a ‘central randomization’ system. Following participant consent, an independent member of the research team not involved in the consent process was contacted and assigned the participant to their treatment group, based on the randomization sequence. The research member informed the participant’s clinical team, and the clinical team allocated the participant a PWP in this condition. Blinding to the intervention for participants was not possible.

### Procedures

NHSTT clinical leads identified PWPs of mixed expertise (newly qualified, qualified, and senior). The PWPs provided informed written consent (electronically), and those identified were randomized to a treatment condition. PWPs randomized to focused CBT attended two half-day workshops, in-person, for training in delivering focused CBT, led by a consultant/professor in clinical psychology and facilitated by a trainee clinical psychologist (see Supplementary Appendix A for a detailed description). Prior to attending the workshops, PWPs completed a questionnaire to rate their confidence in delivering CBT for panic disorder. Post-trial, they evaluated the workshops and elements of focused CBT (see Supplementary Appendix B). PWPs in focused CBT only delivered focused CBT, and PWPs in TAU only delivered TAU. Following the workshops, participant recruitment began.

Participants were identified within their NHSTT service following usual assessment procedures, where a main problem descriptor was derived. This assessment covered the inclusion and exclusion criteria outlined in Section ‘Participants’ and four out of five baseline assessment questionnaires were completed (usual service assessment procedure). Eligible participants were invited to take part by their clinical team. The eligible participant was then contacted by a research assistant or the chief investigator to explain the trial. One exclusion criterion was not covered in the standard NHSTT assessment (involvement in another research project); therefore, the research team checked this with participants. If eligible, the information sheet was provided, questions were answered, and informed consent was completed electronically, via Microsoft Forms. Participants then completed an additional self-report questionnaire online, which was their final baseline assessment questionnaire before randomization (panic safety-seeking and approach-supporting behaviors questionnaire (P-SSASBQ)). After randomization, participants were allocated a PWP in the appropriate condition by their clinical team, followed by a ‘treatment planning session’ to confirm their main problem and provide an overview of treatment. Treatment began following this, and participation ended after the final treatment session and outcome measures consistent with NHSTT practice.

### Interventions

Table 1 provides a summary of each intervention, including the content, supervision provided, and the similarities/differences between interventions. TAU involved either cCBT (*n* = 10) or GSH (*n* = 14), and following randomization to TAU, the treatment received was chosen after discussion with a PWP, in line with usual procedure. cCBT is a low-intensity intervention delivered through ‘SilverCloud’ platform. After a ‘set-up’ call with a PWP on the program called ‘Space from Panic’, individuals worked through the seven modules themselves, having reviews with a PWP. In contrast, after the treatment planning session, GSH involved six to eight treatment sessions lasting 30 minutes. Focused CBT involved six to eight 30-minute treatment sessions, delivered by qualified PWPs trained as previously described.

**Table 1.** Intervention description, supervision provided, and similarities and differences

Intervention	Description	Supervision	Similarities and differences between interventions
TAU GSH	GSH involved an initial consultation with a PWP lasting up to 45 minutes, followed by six to eight treatment sessions lasting 30 minutes (weekly treatment for one service and fortnightly for the other, as this was their usual procedure). PWPs guided the participant through low-intensity CBT techniques, using self-help materials provided in between sessions. Content included psychoeducation about the panic cycle and fight or flight response, exposure to feared situations until individuals become used to the feared situations (habituation using a graded hierarchy), psychoeducation of SSBs and how they maintain panic disorder, worry management, distraction techniques, and breathing exercises. Skills and techniques taught varied according to individual panic disorder presentations.	Usual service supervision, which involved both individual (1 hour, weekly) and group supervision (one and a half hours, fortnightly).	Both treatments (TAU and Focused CBT) explored safety-seeking behaviors’ role in panic disorder, involved exposure to feared situations and materials between sessions were provided. However, in focused CBT, the formulation focused on Clark’s (1986) vicious panic cycle, whereas in TAU a general ‘five areas formulation’ was used (situation, thoughts, emotions, behaviors, and physical sensations) (Padesky & Mooney, 1990). Focused CBT treatment was underpinned by belief disconfirmation and the generation of alternative non-catastrophic misinterpretations, whereas in TAU, treatment involved hierarchies and graded exposure (based on habituation). Therefore, TAU was based on helping one become used to their fearful situations and responses, not altering one’s beliefs about their panic cycle. Also, ASBs were identified and used in focused CBT but not in TAU.
TAU cCBT	cCBT is used to deliver low-intensity interventions using a platform called SilverCloud and involves an initial ‘set up’ call with a PWP lasting up to 20 minutes, which explains the program, called ‘Space from Panic’. It included seven modules (see Supplementary Appendix C for a detailed description), and participants worked through the modules themselves. This typically lasted 6 weeks for one service (one module per week) and 12 weeks for another service (one module, fortnightly). Following the initial call, participants had up to 6 online reviews with PWPs. Participants were offered a final telephone review call to discuss alternative support if needed or to be discharged. SilverCloud was accessed via internet browser or in the app, and participants created their own username and password.	Usual service supervision, which involved both individual (1 hour, weekly) and group supervision (one and a half hours, fortnightly).	
Focused CBT	This involved six to eight 30-minute treatment sessions, delivered by qualified PWPs trained as previously described, delivered either remotely or face-to-face. Focused CBT was delivered weekly in one service, and fortnightly in the other, congruent with service procedures (see Supplementary Appendix D for a session-by-session guide). As focused CBT was a form of guided self-help (which follows NICE (2020) guidelines for panic disorder) before sessions one to five, a workbook module was provided, introducing the session topics through exercises participants completed before the session (see Supplementary Appendix E for an overview of workbooks). Participants brought their completed workbook to the session, and the PWP reviewed the materials, focusing on topics/skills that the participant may have found difficult. No workbook module was given before session six, as this was the final treatment review session.	PWPs in focused CBT attended group clinical supervision via MS Teams, facilitated by the research team, lasting up to 2 hours. It occurred fortnightly for the first 3 months of the trial, stepping down to once a month for the remainder. This involved case discussions/queries to aid PWPs with their treatment sessions.	

### Outcomes

The details of the trial and outcome variables are as specified in the trial pre-registration. Table 2 outlines primary and secondary measures. The Panic Disorder Severity Scale (PDSS) was our primary measure assessing panic symptom severity, and all other measures were secondary. The P-SSASBQ was developed as a secondary aim for this trial and involved adding seven ASBs that may commonly be used in the treatment of panic disorder to the original panic SSB questionnaire. Therefore, this was the first time this measure has been used. The items were developed in consultation with a small panel of experts, with feedback guiding both the inclusion and refinement of items.

**Table 2.** Primary and secondary outcome measures

Measure	Characteristics	Timepoints
Panic Disorder Severity Scale (PDSS) (Shear et al., 1997).	A seven-item measure assessing panic attack and panic symptom severity over the last week. Higher scores indicate greater panic severity. It has been found to have excellent inter-rater reliability, excellent internal consistency (*α* = .92) (Houck, Spiegel, Shear, & Rucci, 2002), and good convergent and discriminant validity (Shear et al., 1997). For this study, it was found to have good internal consistency (*α* = .82).	Administered at baseline (prior to treatment session one) and at every treatment session. Self-report through the NHSTT electronic portal.
Patient Health Questionnaire for depression (PHQ–9) (Kroenke, Spitzer, & Williams, 2001).	A nine-item measure assessing depression. Scores range from 0(not at all) to 3(nearly every day). Total scores range from 0 to 27, and higher scores indicate greater depression severity. It has been shown to have a high degree of internal consistency (*α* = .88) and test–retest reliability (Zuithoff et al., 2010). For this study, it was found to have good internal consistency (*α* = .85).	Administered at baseline (prior to treatment session one) and at every treatment session. Self-report through the NHSTT electronic portal.
Generalized Anxiety Disorder scale (GAD–7) (Spitzer, Kroenke, Williams, & Löwe, 2006).	A seven-item measure asking people to rate how bothered they have been over the last 2 weeks by common anxiety difficulties. Answers range on a scale from 0(not at all) to 3(nearly every day), with higher scores indicating higher generalized anxiety. It has shown to have excellent reliability (*α* = .92) and good validity (Spitzer et al., 2006). For this study, it was found to have good internal consistency (*α* = .88).	Administered at baseline (prior to treatment session one) and at every treatment session. Self-report through the NHSTT electronic portal.
Work and Social Adjustment Scale (WSAS) (Mundt, Marks, Shear, & Greist, 2002),	A brief five-item measure that assesses how one’s problem(s) impact their day-to-day life and functioning. Scores range from 0(not at all) to 8(very severely), with higher scores indicating greater impairment. It has shown to have good reliability (*α* = .82) and validity (Mundt et al., 2002). For this study, it was found to have good internal consistency (*α* = .82).	Administered at baseline (prior to treatment session one) and at every treatment session. Self-report through the NHSTT electronic portal.
Panic Safety-Seeking and Approach-Supporting Behavior Questionnaire (P-SSASBQ).	A modified version of the panic safety-seeking behavior questionnaire (Salkovskis et al., 1996). A 22-item measure, with 15 items focused on safety-seeking behaviors and seven items identifying approach-supporting behaviors. Scores ranged from ‘never’ to ‘always’ (see Supplementary Appendix G). Internal consistency for the 15 items of safety-seeking behaviors was *α* = .66 and for the seven approach supporting behaviors items *α* = .49.	Administered at baseline, mid-treatment, and end of treatment as self-report through Microsoft Forms.

### Statistical analysis

To determine our sample size, we conducted a G*Power priori analysis employing a mixed-design analysis of variance. Based on a Cohen’s *f* effect size of 0.25 (moderate) with 90% power at an *α* level of 0.05, two groups and two measurements, a total sample size of 46 participants was required.

An intention-to-treat (ITT), mixed model ANOVA was used for the primary aim/hypothesis. The model included a within-subjects variable (timepoint) and between-subjects variable of treatment (focused CBT vs TAU), with the dependent variable as panic severity (measured by the PDSS). Where appropriate, follow-up tests were performed to determine whether changes in the dependent variable were due to one of the independent variables (timepoint or treatment). The same ITT mixed model was used for secondary outcomes of depression, anxiety, and WSAS.

A multiple linear regression was used for our exploratory aim. For the first model, change in panic severity was the dependent variable, and ASB/SSB at the start of treatment were the independent variables with treatment (focused CBT vs TAU) as the dummy variable. For the second model, ASB/SSB at the end of treatment were the independent variables, and all other variables remained the same.

NHSTT also calculates recovery rates, which are defined as a case who starts in ‘clinical caseness’ on a specific measure at the start of treatment and not in ‘clinical caseness’ at the end of their treatment. ‘Reliable improvement’ was also calculated, taking the difference between one’s first and last scores; for the PDSS, this was ≥5.

### Funding source

There was no funding source for this trial.

### Treatment fidelity

We defined treatment fidelity as the extent to which the treatment was implemented as intended (Borrelli, 2011; O’Shea, McCormick, Bradley, & O’Neill, 2016; Sanetti, Cook, & Cook, 2021). We modified the revised cognitive therapy scale (CTS-R) (Blackburn et al., 2001; Liness et al., 2021) to assess for treatment fidelity to ensure it reflected ‘Step Two’ lower intensity intervention (see Supplementary Appendix F). Two authors (SA and PS) independently rated 10% of the total sample of the therapy recordings (*n* = 12), and inter-rater reliability was calculated, revealing ‘almost perfect agreement’ (Cohen’s kappa = .89, *p* < .001). Following this, SA rated 25% of all therapy recordings, randomly selected, and all PWPs in focused CBT were assessed.

## Results

### Participant recruitment

Participants were recruited between January 2024 and August 2024. Eighty-eight patients were assessed for eligibility at triage assessment, of whom 72 consented to participate and were randomly allocated in a 1:1 ratio (36 in each treatment). The triage assessment helped identify a ‘problem descriptor’ of panic (standard NHSTT procedure), and once this was established, consent and randomization were initiated. Following randomization, participants had a treatment planning session prior to their first treatment session (standard practice for NHSTT). During treatment planning, which involves a more detailed assessment of the main problem, it was identified that for 12 participants, panic disorder was not the primary problem, resulting in exclusion. This suggests a limitation with the triage assessment process (discussed in Section ‘Limitations and future research’).

**Figure 1.**
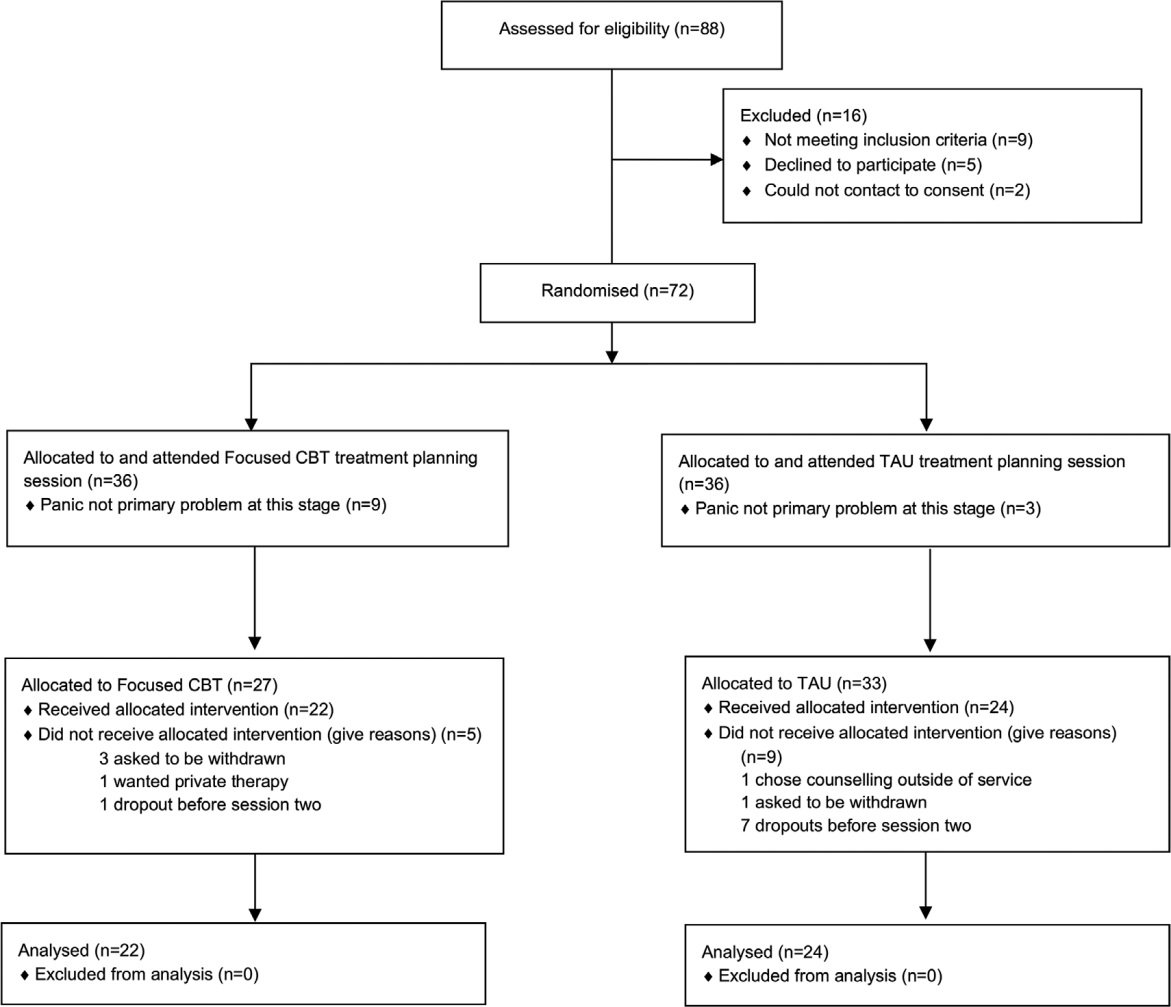
CONSORT flow diagram.

Of those 60 participants, 27 (45%) remained in focused CBT and 33 (55%) in TAU. Of those in focused CBT, 22 received the intervention, and of those who were randomly assigned to TAU, 24 received the allocated intervention, defined as completing the first treatment session, resulting in a total of 46 participants, all included in the ITT analysis (Figure 1).

### Participant characteristics

Overall, the 46 participants had a mean age of 35.9 years (*SD* = 11.7), ranging from 18 to 67 years and those in focused CBT were somewhat younger than those in TAU but not significantly different (*p* > .05). The final sample consisted of 31 females (67.4%) and 15 males (32.6%), with 37 being from a white British background (80.4%). The majority of participants were taking medication (58.7%, *n* = 27), with 41.3% (*n* = 19) taking no medication at all. Those in focused CBT reported non-significantly higher levels of depressive and general anxiety symptoms compared to those in TAU, but reported significantly higher levels of their difficulties impacting their daily life and functioning from the WSAS (*p* = .04) (see Table 3).

**Table 3.** Participant demographics

Demographic	Focused CBT (*n* = 22)	TAU (*n* = 24)	Total (*n* = 46)
*Gender: n*(%)			
Female	14 (63.6%)	17 (70.8%)	31 (67.4%)
Male	8 (36.4%)	7 (29.2%)	15 (32.6%)
*Ethnicity*			
White British	18 (81.8%)	19 (79.2%)	37 (80.4%)
Asian British	1 (4.5%)	1 (4.2%)	2 (4.3%)
Mixed	1 (4.5%)	1 (4.2%)	2 (4.3%)
Other	1 (4.5%)	1 (4.2%)	2 (4.3%)
Black British	0 (0%)	1 (4.2%)	1 (2.2%)
Asian Other	0 (0%)	1 (4.2%)	1 (2.2%)
Unknown	1 (4.5%)	0 (0%)	1 (2.2%)
*Medication*			
Propranolol	6 (27.3%)	8 (33.3%)	14 (30.4%)
SSRIs	5 (22.6%)	4 (16.7%)	9 (19.6%)
Other	4 (18.1%)	0 (0%)	4 (8.7%)
None	7 (31.8%)	12 (50%)	19 (41.3%)
*Age: M (SD)*	32.5 (11.3)	39.1 (11.4)	35.9 (11.7)
*Depressive symptoms (PHQ–9)*	13.82 (7.34)	11.50 (6.06)	12.61 (6.73)
*Generalized anxiety (GAD–7)*	16.18 (4.87)	13.25 (5.10)	14.65 (5.15)
*Daily functioning (WSAS)*	20.43 (8.80)	14.83 (9.12)	17.44 (9.30)^a^

Abbreviations: SSRIs, selective serotonin reuptake inhibitors.

a Significant at p = .04.

### Primary outcome

**Figure 2.**
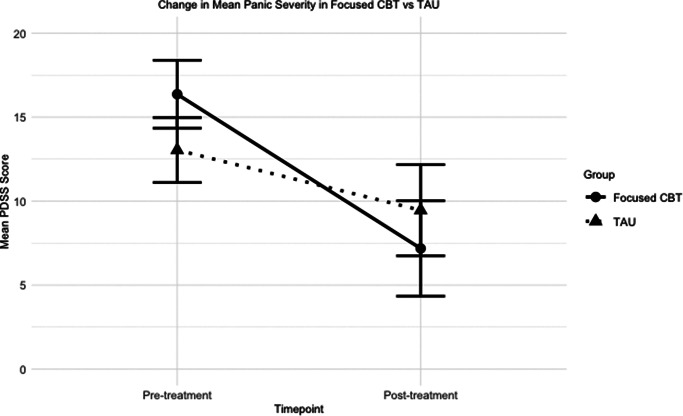
Change in mean PDSS score pre to post treatment in focused CBT vs TAU.

The results for the primary and secondary outcomes are shown in Table 4. We conducted a mixed-model ANOVA on our primary outcome measure (PDSS assessing panic severity). The ANOVA revealed a significant main effect of timepoint *F*_(1,44)_ = 46.73, *p* < .001, *ηp*^2^ = 0.515. There was no main effect of group *F*_(1,44)_ = 0.14, *p* = 0.71. These effects were modified by a significant group-timepoint interaction *F*_(1,44)_ = 8.99, p = 0.004, *ηp*^2^ = .170 (see Figure 2). Follow-up t-tests showed a significant between-group difference at baseline *t*(44) = −2.40, *p* = 0.02, and the post-treatment difference was not significant *t*(44) = 1.16, *p* = 0.25. Given this pattern of results and the presence of a significant interaction, we conducted an analysis of covariance with PDSS post-treatment scores as the dependent variable and baseline PDSS (pre-treatment) scores as a covariate. This resulted in a significant main effect of group *F*_(1,43)_ = 4.93, *p* = 0.03. This suggested that, allowing for baseline differences, those in focused CBT reported significantly lower levels of panic severity post-treatment compared to those in TAU (focused CBT adjusted *M* = 6.15, TAU adjusted *M* = 10.40).

**Table 4.** Outcome measures mean (SD) scores

Measure	Focused CBT (*n* = 22)		TAU (*n* = 24)	
	*M*	(SD)	*M*	(SD)
PDSS baseline	16.36	4.36	13.04	4.97
PDSS post	7.18	6.44	9.46	6.83
PHQ–9 baseline	13.82	7.34	11.50	6.06
PHQ–9 post	8.32	7.54	7.71	6.92
GAD–7 baseline	16.18	4.87	13.25	5.10
GAD–7 post	8.05	5.70	8.42	6.94
WSAS baseline	20.43	8.80	14.83	9.12
WSAS post	12.10	10.73	12.42	11.70
SSBs baseline	1.46	0.43	1.33	0.32
SSBs post	1.14	0.64	1.23	0.36
ASBs baseline	1.18	0.41	1.06	0.42
ASBs post	1.53	0.45	1.71	0.49

Applying NHSTT rules to calculate the recovery rates in terms of PDSS scores, we found a 73% recovery rate in focused CBT compared to 35% in TAU, suggesting a significantly higher rate of recovery in panic severity in the focused CBT group (χ^2^ (1, *n* = 46) =6.02, *p* = 0.029). Reliable improvement on the PDSS was also calculated. We found 77.3% of those in focused CBT reliably improved compared to 41.7% in TAU (χ^2^ (1, *n* = 46) =6.002, *p* = 0.019), suggesting a significantly greater improvement in panic severity in focused CBT.

### Secondary outcomes

#### Depression

The mixed-model ANOVA revealed a significant main effect of timepoint *F*_(1,44)_ = 27.61, *p* < .001, *ηp*^2^ = 0.386. However, there was no significant main effect of group *F*_(1,44)_ = 0.62, *p* = .43, and there was no significant group-timepoint interaction *F*_(1,44)_ = 0.93, *p* = .34.

#### Generalized anxiety

The mixed-model ANOVA revealed a significant main effect of timepoint *F*_(1,44)_ = 49.34, *p* < .001, *ηp*^2^ = 0.529. However, there was no significant main effect of group *F*_(1,44)_ = 0.82, *p* = .37, and there was no significant group-timepoint interaction *F*_(1,44)_ = 3.20, *p* = .08.

#### Daily functioning (WSAS)

The mixed-model ANOVA revealed a significant main effect of timepoint *F*_(1,43)_ = 11.06, *p* = .002, *ηp*^2^ = 0.205. However, there was no significant main effect of group *F*_(1,43)_ = 1.05, *p* = .31, and there was no significant group-timepoint interaction *F*_(1,43)_ = 3.35, *p* = .07.

### Exploratory aim

To test our exploratory aim, we conducted a multiple linear regression to test whether SSBs and ASBs predicted change in panic severity. We found the level of ASBs and SSBs at the start of treatment did not predict a change in panic severity, *F*_(4,41)_ = 2.43, *p* = .06. However, when examining the level of SSBs and ASBs at the end of treatment, the model was statistically significant, *F*_(4,31)_ = 4.23, *p* = .008, R^2^ = .269, R^2^change = .087. The first block (age) added 2.8% variance, the second block (age, SSBs, and ASBs at the end of treatment) added 19.7% variance, and the final block (age, SSBs, and ASBs at the end of treatment, treatment allocation) added 27% of the variance. The level of SSBs at the end of treatment significantly predicted changes in panic severity *p* = .002. The level of ASBs at the end of treatment did not predict changes in panic severity (*p* = .71).

### PWPs feedback for focused CBT

#### Prior to focused CBT training

PWPs rated their confidence in delivering CBT for panic disorder prior to training. On a scale from 0 to 100 (0 = not confident at all, 100 = extremely confident), it was rated moderately for both delivering CBT for panic disorder in person (*M* = 70, SD = 11.5) and via MS Teams (*M* = 66, SD = 13.9). Confidence in delivering CBT for panic disorder with agoraphobia was rated lower for both in-person (*M* = 55, SD = 16.2) and via MS Teams (*M* = 52, SD = 16).

#### After trial completion

PWPs re-rated their confidence post-trial. PWPs confidence in delivering CBT for panic disorder improved significantly for both in-person *t*(4) = −.26, *p* = .04 (*M* = 84, SD = 8.2) and MS Teams delivery *t*(4) = −3.9, *p* = .018 (*M* = 87, SD = 4.5). Confidence also improved but not significantly for delivering CBT for panic with agoraphobia, in-person *t*(4) = −1.68, *p* = .17 (*M* = 76, SD = 14.7). However, it did improve significantly for MS Teams delivery *t*(4) = −3.73, *p* = .02 (*M* = 81, SD = 2.2).

PWPs evaluated focused CBT elements (workbooks, developing formulations, behavioral experiments, etc.), training, and supervision. On a scale from 0 to 10 (0 = not helpful at all, 10 = extremely helpful), PWPs found the training (*M* = 8.2, SD = 1.3), clinical supervision (*M* = 8.8, SD = 0.8), and patient workbooks (*M* = 8.8, SD = 1.1) very helpful. On a scale from 0 to 10 (0 = not useful at all, 10 = extremely useful), PWPs found the training very useful in helping them aid patients with developing a personalized formulation (*M* = 8.4, SD = 1.1), using discussion techniques to challenge misinterpretations (*M* = 8.4, SD = 1.1), engaging with personalized behavioral experiments involving exposure (*M* = 7.8, SD = 1.7), using individualized ASBs within experiments (*M* = 8.4, SD = 1.1) and developing relapse prevention strategies (*M* = 7.6, SD = 1.1).

Qualitative feedback indicated areas of improvement, such as providing more detail on each treatment session content during training and making workbooks more succinct. Strengths included the usefulness of videos and role plays during training, how the workbooks reinforced learning for patients, and the benefit of patients receiving the workbooks prior to each session to learn techniques and come prepared. Supervision was valued, enabling PWPs to share reflections on cases, learn from peers, and discuss challenging cases.

### Treatment fidelity results

The modified CTS-R included 15 items; each item was rated out of six (see Supplementary Appendix F). Scores of three or above indicated good competence in skills by PWPs (agenda setting, using the workbooks, behavioral experiments used, etc.). Twelve items had an average rating of three or above, suggesting a good level of competence across the majority of skills rated. This suggests PWPs in focused CBT demonstrated proficiency in applying relevant techniques.

Adherence to session guides was rated out of six (excellent adherence). The mean was 4.3, suggesting ‘good’ adherence to session guides. The mean overall skill rating in how PWPs applied focused CBT (zero = poor, six = excellent) was four, suggesting a ‘good’ level of skill. Mean patient difficulty was rated as one, indicating low difficulty.

## Discussion

This trial’s primary aim was to evaluate the efficacy of a focused low-intensity CBT intervention for panic disorder, delivered by PWPs in NHSTT, compared to what is currently delivered (TAU). We found that focused CBT resulted in a significantly greater decrease in panic symptoms at post-treatment compared to TAU (GSH and cCBT), supporting our main hypothesis. These results are consistent with the higher recovery rates noted in this study for focused CBT. However, symptoms of general anxiety, depression, and the impact one’s problems have on one’s daily life and functioning were not significantly different between groups, therefore not consistent with our secondary hypothesis. Both groups showed a significant decrease from pre- to post-treatment across all secondary measures. For our exploratory aim, we note that the measure of ASBs did not have good internal consistency and did not predict change in panic severity; SSBs at the end of treatment were, however, a good predictor.

These findings are consistent with previous research, most notably, Clark et al. (1999) study, which found that brief focused CBT delivered in five to six sessions with self-help modules given before each session (the present modules being a development of these), was as effective in reducing panic symptoms as full treatment. Our findings build on this research as focused CBT was delivered here at low intensity by PWPs, whereas the therapists in Clark et al. (1999) study were cognitive therapist specialists. These findings also align with Grey et al. (2008) study, which found that when therapists were trained in cognitive therapy focusing on Clark’s (1986) cognitive model of panic disorder, significant improvements in treatment for panic disorder were noted. The findings support previous research showing that identifying catastrophic misinterpretations of bodily sensations and safety-seeking behaviors and targeting them within treatment using belief disconfirmation results in greater improvements in panic when compared to exposure-based habituation (Salkovskis et al., 1999, 2007).

In terms of scalability, the brief training workshop was well received by PWPs, enhancing their confidence in applying CBT for panic disorder. The training helped them feel more confident in developing personalized formulations, using behavioral experiments, and developing relapse prevention strategies. They found the workbook modules for participants highly useful and rated the additional supervision received favorably. Overall, it suggests that low-intensity focused CBT, using workbook modules, may be feasible in NHSTT services.

### Limitations and future research

There are several limitations to the present study: (1) The small sample (*n* = 46) limited generalizability; therefore, these results need to be confirmed in a larger scale study. However, statistical power was achieved as set out in the pre-registration. (2) There were differences at baseline between the two groups; however, this was accounted for during statistical analyses. (3) It was necessary to exclude several participants at the treatment planning stage, as it became clear that panic was not the main problem (initial triage is typically relatively brief in Talking Therapy services, conducted over the telephone by PWPs, with the final diagnostic decision being made at the treatment planning stage for all participants). (4) No follow-up was conducted; therefore, we do not know the long-term impact of focused CBT. However, a recent systematic review did find that sustained improvements following treatment for panic disorder are dependent on cognitive change occurring (Aslam et al., 2024), and this was the primary mechanism of change in focused CBT. (5) The ASB scale has its limitations as it was developed in consultation with a small panel of experts and has a limited developmental history. However, the use of this scale was exploratory. (6) Finally, an external assessor did not rate a subset of the therapy recordings. However, ethical guidelines were followed with approval of only the research team members who were employed by the NHS trust to view and assess therapy recordings. Nonetheless, to help strengthen treatment fidelity assessment, the trial may have benefited from an external assessor.

Future research should investigate the impact of low-intensity focused CBT for panic disorder across multiple NHSTT sites. Conducting 6- and 12-month follow-ups and comparing these with TAU can also help evaluate long-term outcomes in panic. It may also be useful for future research to collect agoraphobic data to determine if there are differences in therapeutic outcomes between those with solely panic disorder and those with panic disorder with agoraphobia. This can help develop focused CBT further, but also aid with service development, to determine if differentiating between panic disorder and panic with agoraphobia would be beneficial for treatment outcomes. Finally, the poor internal consistency and predictive role of the P-SSASBQ suggest that developing a more robust measure of SSBs and ASBs would be useful, and having clinicians administer it may help individuals differentiate between safety-seeking vs approach-supporting. Due to the limitations of this scale and the need for a greater developmental history, a Delphi study to help develop the ASB scale would be helpful for research and clinical practice.

### Theoretical and clinical implications

These findings are consistent with the cognitive theory of panic disorder (Clark, 1986), which underpins focused CBT in general and the specific details of both workbooks and treatment offered here. The theory emphasizes the importance of identifying catastrophic misinterpretations of bodily sensations and SSBs, reducing these within treatment, and developing alternative, less threatening interpretations of bodily sensations. The effectiveness of focused CBT in this study highlights the importance of applying the cognitive approach within treatment.

Given these findings, clinical implications arise: (1) The importance of a cognitive focus in treating panic disorder. Although TAU resulted in improvements, a major difference was the primary focus on catastrophic misinterpretations in focused CBT and using behavioral experiments to disconfirm beliefs. However, TAU’s focus was on graded exposure using habituation. Although there was some cognitive focus within TAU, this involved general ‘Negative Automatic Thoughts’, rather than panic misinterpretations. (2) The findings support the use of this lower-intensity CBT for panic disorder in NHSTT, incorporating workbook modules. (3) Although face-to-face therapy was an option, all participants completed treatment remotely via MS Teams, suggesting the treatment’s feasibility for remote delivery. (4) The training methods used in focused CBT, as evaluated in this trial, add support for the methodology of delivering brief training with expert supervision and can therefore be applied in other areas. For example, in low- and middle-income countries (LMIC), using this model within focused CBT training, of having a brief training period with case discussions, role plays, vignettes, video recordings, and providing regular expert supervision while psychological therapists are implementing the teaching may help to train psychological therapists on a larger scale. Then, examining the impact of this training model on patient outcomes would be helpful. This supports models and reviews that have proposed and examined this method of training in LMIC (Beck, Nadkarni, Calam, Naeem, & Husain, 2016; Singla et al., 2017). (5) An important clinical implication involves the use of panic disorder-specific measures in assessment and patient outcomes. Although no significant differences were observed in general mental health measures (PHQ-9, GAD-7, and WSAS) between the two groups here, the significant difference in the panic-specific measure (PDSS scores) and higher recovery rates in focused CBT on the PDSS highlight the importance of using panic-specific outcome measures to help capture change and specific outcomes. Also, using panic disorder-specific measures within assessment may help gather important information regarding specific panic presentations and potential treatment targets within therapy. (6) Finally, due to the number of initial assessments of patients found not to have panic disorder as their primary difficulty, enhanced training and workshops on disorder-specific assessment would be beneficial for improving diagnostic accuracy and, ultimately, personalizing treatment for patients.

## Conclusions

Overall, findings suggested that focused CBT was significantly more effective in reducing panic symptoms in individuals with panic disorder compared to TAU (GSH and cCBT). This supports the use of low-intensity CBT treatment for panic disorder, focused on the cognitive theory of panic disorder, using workbook modules to improve symptoms of panic, compared to treatment focused on graded exposure based on habituation.

## Supporting information

Aslam et al. supplementary material Aslam et al. supplementary material

## Supplementary material

The supplementary material for this article can be found at http://doi.org/10.1017/S0033291725102353.
