# Supplementary material for: Evaluating the effectiveness of a focused CBT training for panic disorder: a randomized parallel trial
Source: Psychol Med. 2025 Nov 24;55:e356. doi: 10.1017/S0033291725102353 (PMC13058618; doi:10.1017/S0033291725102353)
Supplement: Aslam et al. supplementary material [file S0033291725102353sup001.docx]

Appendix A - Description of training workshops for PWPs in focused CBT

| Day | Component | Description |
| --- | --- | --- |
| 1 | **Pre-workshop survey** | Four question survey was provided to all PWPs to complete before the training workshops began to assess their confidence in delivering CBT for panic disorder. |
| 1 | **Trial explanation** | Rationale and scientific background for the trial provided. |
| 1 | **Cognitive approach to anxiety** | Theory provided on what the cognitive approach is to understanding and treating anxiety disorders |
| 1 | **Panic and agoraphobia description** | Description of what panic attacks are and how this is different to panic disorder. Signs and symptoms of panic attacks outlined. Agoraphobia explained and its comorbidity with panic disorder. Typical believes in panic attacks and in agoraphobia provided. |
| 1 | **Cognitive theory of panic disorder and maintenance** | Clark (1986) model of panic disorder explained (panic vicious cycle). Factors maintaining panic disorder discussed including safety-seeking behaviours and selective attention to bodily cues. |
| 1 | **Safety-seeking and approach supporting behaviours** | Explanation of how safety-seeking behaviours differs to approach supporting behaviours. Examples of both were provided within the context of panic disorder. |
| 1 | **Brief outline of CBT for panic disorder** | Identifying catastrophic misinterpretations of bodily sensations, generating alternative non catastrophic interpretations of bodily sensations and finally, testing out the validity of catastrophic and non-catastrophic interpretations of bodily sensations using discussion and behavioural experiments. |
| 1 | **Beginning treatment: Assessment/formulation with video examples and role plays** | This involved explaining to the PWPs how to develop an individualised formulation (Clark, 1986 model) with the panic patient through identifying a recent panic attack episode and deriving the specific bodily sensations and catastrophic misinterpretations. Examples of specific links between bodily sensations and catastrophic misinterpretations were given (for example heart racing means I am having a heart attack).  Example key questions were provided to help PWPs with obtaining this information during this phase of treatment. Questions given were aimed to help PWPs elicit bodily sensations, catastrophic misinterpretations and the emotions of the patient.  Video clips were shown to PWPs of a consultant clinical psychologist deriving a panic vicious cycle.  Role plays were used to give PWPs a chance to practice developing a formulation. |
| 1 | **Assessing for safety-seeking behaviours** | This involved teaching PWPs how to identify the specific safety-seeking behaviours panic patients are engaging with which may maintain their panic vicious cycle. Example questions were provided to help elicit this. Explicit reference was made for PWPs to link the safety-seeking behaviours back to the formulation.  Video clips shown of a consultant clinical psychologist identifying safety-seeking behaviours linked to the individual’s formulation. |
| 1 | **Use of discussion techniques (theory A vs theory B)** | Helping PWPs learn different techniques to elicit the current belief, theory A (catastrophic misinterpretations) and how they use discussion to develop and build evidence of a new belief, theory B. An example includes PWPs exploring the patient’s bodily sensation that occurs when excited vs when panicky and what the difference between the two are (i.e. there is no difference in sensations but the interpretation of the sensations).  Encouragement provided to use belief ratings to help monitor change in beliefs. Metaphors were provided to aid with this. |
| 2 | **Panic diaries** | Introducing PWPs to the panic diaries to be used with patients. This involved providing copies of the three different panic diaries and showing PWPs how they explain the panic diaries to patients, including identifying bodily sensations (panic diary 1), catastrophic misinterpretations with a belief rating (panic diary 2) and the alternative belief developing (panic diary 3). |
| 2 | **Behavioural experiments** | Understanding what behavioural experiments are and how they are used in treatment for panic disorder. Provided PWPs with an overview of the purpose for using behavioural experiments during treatment.  Discussed the differences between behavioural experiments and exposure. |
| 2 | **Behavioural experiments with video clips** | Outlined the components of a behavioural experiment and how to link this to a patient’s formulation (situation, belief/prediction and rating, experiment, outcome and learning).  Example behavioural experiments for specific panic beliefs were shown.  Video clips were shown to PWPs of a consultant clinical psychologist planning and completing different behavioural experiments. |
| 2 | **Using approach supporting behaviours in behavioural experiments** | Examples were provided to PWPs on how they can identify and use approach supporting behaviours during behavioural experiments to help manage progression in experiments. |
| 2 | **Behavioural experiment record sheet with role plays** | Provided a copy of and went through the behavioural experiment record sheet.  Role play for PWPs to develop their own behavioural experiments using the record sheet with specific panic beliefs. |
| 2 | **Working with common panic beliefs and problem solving** | Provided PWPs with examples of how to work with specific panic beliefs (catastrophic misinterpretations of bodily sensation) and how behavioural experiments can be developed to work on these beliefs.  Strategies provided to PWPs on what to do if belief is not changing through the completion of behavioural experiments. |
| 2 | **Therapy blueprint** | PWPs guided through different components of developing a panic therapy blueprint. Step by step breakdown of how to create this. |
| 2 | **Workbook module guide** | Introduction to the five workbook modules and the topics that are covered in the workbook modules. Instructions provided to PWPs on when to provide them and how to use them during the focused CBT sessions. |
| 2 | **Focused CBT session by session guide** | A detailed overview was provided to PWPs about each focused CBT session. This included what materials would be needed prior to the session, what materials need to be provided to the patient after the session, what topics may be explored in each session and how they will use the completed workbooks during the focused CBT sessions. |

Appendix B – PWP Survey (pre and post-trial)

**Effectiveness of focused CBT Training for Panic Disorder**

Pre and Post:

**Confidence in CBT (0 = not at all confident, 100 = extremely confident)**

1. How confident are you in delivering CBT for panic disorder with agoraphobia?
2. How confident are you in delivering CBT for panic disorder without agoraphobia?
3. How confident are you in engaging with CBT for panic disorder with agoraphobia via MS Teams?
4. How confident are you in engaging with CBT for panic disorder via MS Teams?

Post:

**Workshop (Helpful scale: 0 = not helpful, 10 = extremely helpful)**

1. How helpful did you find the training workshops? (0-10)
2. Any specific comments?

**Supervision**

1. How helpful did you find the additional supervision sessions provided during the project? (0-10)
2. Any specific comments?

**Client materials**

1. How helpful did you find the provision of additional reading materials for client? (0-10)
2. What are your thoughts on the materials provided?

**Components of Training (0 = not at all useful, 10 = extremely useful)**

1. How useful did you find the training in terms of helping patients do the following:
   1. Formulation and shared understanding (vicious circle)
   2. Discussion techniques to challenge catastrophic misinterpretations
   3. Engage with behavioural experiments involving exposure
   4. The use of approach supporting behaviours to facilitate behavioural experiments
   5. Relapse prevention strategies

Appendix C – cCBT Modules Description

| Space from Panic Module | Description |
| --- | --- |
| Module 1  Getting Started | Introduction to SilverCloud and the space from panic module. Explains how CBT can help, helps develop a personalised ‘five areas’ cognitive cycle, provides personal stories of people with panic and their journey and emphasises the importance of daily practice. |
| Module 2  Understanding Panic | Normalises experience of panic, explains symptoms of panic, personal stories of panic to normalise it, ‘staying in the present’ body scan and summary of the module. |
| Module 3  Noticing Feelings | Explains how emotions can impact thoughts and behaviours, provides an overview of understanding emotions and physical changes and how they can be impactful, helping people change their physical sensations to improve mood, overview of how small changes to lifestyle choices can impact wellbeing (sleep, alcohol, diet, caffeine, physical activity and medication), ‘staying in the present’ PMR exercise and summary of module. |
| Module 4  Facing your Fears | Introduction to habituation using graded exposure, explaining the role of avoidance and safety-seeking behaviours on anxiety with specific examples for panic, developing a hierarchy ladder for graded exposure, recording and reviewing exposure tasks, how to put exposure into action (graded, repeated, continuous, without distraction) and summary of module. |
| Module 5  Spotting and Challenging Thoughts | Introduces the role of thoughts and their impact on mood. Examines general unhelpful thinking styles, how to ‘catch’ thoughts, focuses on individual ‘five areas’ CBT cycle, creating evidence for and against thoughts, ‘staying in the present’ relaxation exercise around watching thoughts and summary of module. |
| Module 6  Managing Worry | Introduces the role of worry and the impact on anxiety, explains the ‘worry cycle’, distinguishes between practical vs hypothetical worries, guides people in creating their own worry tree and problem solving, identifies how to manage worry using ‘worry time’ and refocusing, ‘staying in the present exercise’ and summary of module. |
| Module 7  Bringing it all Together | Summarises learning from previous modules, helps person identify warning signs and how to manage them, provides space for people to create further goals, completion of another relaxation exercise, personal stories about the importance of continued practice and summarising the module. |

Appendix D – Focused CBT Session by Session Guide

| Session |  | Component | Description |
| --- | --- | --- | --- |
| 1 |  | Agenda setting | Collaboratively develop an agenda with the patient. Ask them about what they would like to discuss based on workbook module 1. |
|  |  | Normalising panic | Discussion about panic attacks and who gets them. The aim of this discussion is to help one see that panic is common, anyone can experience panic attacks and it does not mean they are ‘weak’ or ‘deficient’. |
|  |  | Psychoeducation | This involves two key parts: (1) Linking bodily sensations to catastrophic misinterpretations and (2) Explaining how therapy can help. |
|  |  | Formulation | A key aim of session one is to ensure the patient develops their personalised version of the panic vicious circle (formulation). This should be completed in workbook 1 before the session, however, some people may have got stuck. Patients may also have doubts about this, so it is important to check this and work through any doubts with them. The goal is to ensure they have their personalised version which will guide treatment.  Go through their formulation with them. You may also use completed versions of ‘Panic Diary 1’ to identify other bodily sensations people experience.  If patients get stuck: guide them through developing their own vicious circle. Identify the recent panic attack situation and trigger, followed by any quick thoughts, followed by any emotions, then sensations, then catastrophic misinterpretations. |
|  |  | Module 2, Panic Diaries 2 and next session | At the end of the session, provide workbook module 2 AND copies of the ‘Panic Diaries 2’. Stress the importance of working through this before their next session. |
| 2 |  | Agenda setting | Collaboratively develop an agenda with the patient. Ask them about what they would like to discuss based on workbook module 2. |
|  |  | Reviewing panic diary 2 | Review completed panic diaries, noticing the ‘thoughts of disaster’ people have managed to identify based on their bodily sensations. |
|  |  | Discussion techniques | The aim of this discussion is to review the three areas of ‘examining the evidence’ patients will have worked through in module 2:  (1) Panic attacks are different to the serious situations people have been comparing them to.  (2) There is not something seriously wrong with the person having panic attacks.  (3) There are alternative explanations for symptoms.  In this part of the session, review the exercises the patient has completed for each of the three points above to aid with discussion. A key point is to examine what the patient has learned after completing each of the three exercises. |
|  |  | Module 3, Panic Diaries 3 and next session | At the end of the session, provide workbook module 3 AND copies of the ‘Panic Diaries 3’. Stress the importance of working through this before their next session**.** |
| 3 |  | Agenda setting | Collaboratively develop an agenda with the patient. Ask them about what they would like to discuss based on workbook module 3. |
|  |  | Reviewing panic diary 3 | Review the ‘answers’ patients are developing to their catastrophic thoughts. |
|  |  | Psychoeducation of safety-seeking AND approach supporting behaviours | **Safety-Seeking**: Patients will have worked through this exercise and may have been able to identify different safety-seeking behaviours they use. Discuss with them the impact they can have. Be sure to use the specific safety-seeking behaviours they have identified.  **Approach supporting:** The workbook will have explained approach supporting behaviours and provided examples. Identify with the patient potential approach supporting behaviours they could use or help develop their own. |
|  |  | Behavioural experiments | A key part of this session is to complete a behavioural experiment. Summarise their panic circle and ensure the behavioural experiment is specific to the individual and their panic circle. When planning, be sure to emphasise dropping safety-seeking behaviours and using approach supporting behaviours. Document this on the behavioural experiment record sheet. Ask what they have learned from experiments completed to help link this back to their vicious circle. Encourage completion of a behavioural experiment in between this session and next session. |
|  |  | Administering additional outcome measure | Administer the panic safety-seeking and approach supporting behaviour measure. |
|  |  | Module 4 and next session | Provide workbook module 4. Stress the importance of working through this before their next session |
| 4 |  | Agenda setting | Collaboratively develop an agenda with the patient. Ask them about what they would like to discuss based on workbook module 4. |
|  |  | Discussion techniques | Review patient’s responses in workbook module 4 to the things they noticed when they dropped safety-seeking behaviours and used approach supporting behaviours. Highlight the positive impact of dropping safety-seeking behaviours and using approach supporting behaviours. |
|  |  | Attention experiment | Discuss the negative impact of focusing one’s attention on symptoms/sensations.  Complete small experiment to test influence of attention if this was not completed at home/they did not understand the attention experiment from the workbook module. |
|  |  | Discussion techniques: Looking for triggers | Use of discussion to help identify different triggers to the patient’s panic attacks. Emphasise the role that emotions can have on triggering panic attacks. Look through the workbook to identify any triggers, in particular emotional reactions the patient was able to identify.  The aim is to help the patient realise that they can have normal emotional reactions which may have resulted in them becoming oversensitive to, which can easily spiral into a panic attack as it can create changes in bodily sensations and therefore frightening thoughts. This realisation may help prevent the patient’s reactions from spiralling into a panic attack. |
|  |  | Module 5 and next session | At the end of the session, you will need to provide workbook module 5. Stress the importance of working through this before their next session. |
| 5 |  | Agenda setting | Collaboratively develop an agenda with the patient. Ask them about what they would like to discuss based on workbook module 5. |
|  |  | Therapy blueprint | Upon patients completing workbook module 5 before the session, patients should have created their own therapy blueprint as part of the workbook instructions. The aim of this session is to review their blueprint and discuss this with them. This will involve: Reflecting on learning points in therapy, developing a plan of how to continue implementing and building on new skills as well as identifying ways to overcome future challenges. |
| 6 |  | Agenda setting | Collaboratively develop an agenda with the patient. |
|  |  | Administering outcome measure | Administer the panic safety-seeking and approach supporting behaviours questionnaire as ‘end’ point. |
|  |  | Recap | Review of content during focused CBT and what they have learnt. |

Appendix E – Focused CBT Workbook Modules Overview

| Workbook | Component | Description |
| --- | --- | --- |
| 1 | **Normalising panic** | Discussion about panic attacks and who gets them. |
|  | **Psychoeducation** | The Cognitive Approach to panic – linking bodily sensations to catastrophic misinterpretations.  Psychoeducation about therapy and how it can help (theory A vs theory B). |
|  | **Formulation** | Developing personalised formulation of vicious cycle of panic model (Clark, 1986). . |
|  | **Panic diary 1** | Helping individuals to record bodily sensations experienced during panic attacks and tracking frequency of panic attacks. |
|  |  |  |
| 2 | **Maintenance of panic psychoeducation** | (1) Looking for evidence, (2) Safety-seeking and avoidance, (3) Attention, (4) Images |
|  | **Panic diary 2** | Helping individuals to record bodily sensations, catastrophic misinterpretations and belief ratings. |
|  | **Examining the evidence for panic attacks** | Examining the evidence:  (1) Panic attacks are different to the serious situations people have been comparing them to.  (2) There is not something seriously wrong with the person having panic attacks.  (3) There are alternative explanations for symptoms. |
| 3 | **Panic diary 3** | Helping individuals to record sensations, catastrophic misinterpretations and answers to these misinterpretations. |
|  | **Psychoeducation of safety-seeking behaviours** | Examining the specific safety-seeking behaviours one engages with to save themselves.  Negative effects of safety-seeking behaviours, making things worse. |
|  | **Psychoeducation of approach-supporting behaviours** | Using behaviours to help one make it easier to confront their fears.  Identifying specific approach supporting behaviours for people to use. |
|  | **Behavioural experiments** | Introduces behavioural experiments as a way of testing specific beliefs. Exercise to ask individuals about specific beliefs and fears they can bring to the session to help with guiding a behavioural experiment. |
| 4 | **Reviewing using approach supporting behaviours and dropping safety-seeking behaviours** | Identifying positive impact of dropping safety-seeking and using approach supporting behaviours. |
|  | **Attention experiment** | Highlighting negative impact of focusing one’s attention on symptoms/sensations. Behavioural experiment to test influence of attention in maintaining panic. |
|  | **Looking for triggers to panic attacks** | Discussion of different triggers for people’s panic attacks. Emphasising role of (1) going back to previous places where panics happened, (2) attending/noticing physical symptoms and (3) how emotional reactions can trigger panic attacks. |
|  | **Behavioural experiment recap** | Going through step-by-step guide of setting up their own behavioural experiment to test their beliefs and worst fears. |
| 5 | **Therapy blueprint** | Reflect on learning points in therapy, developing a plan of how to continue implementing and building on new skills as well as identifying ways to overcome future challenges. |

Appendix F – Modified CTS-R

**LOW INTENSITY FOCUSED CBT FOR PANIC (STEP 2 TREATMENT):**

**CHECKLIST OF THERAPIST COMPETENCY**

**The Rating of the Scale**

The present seven-point scale (i.e. a 0-6 Likert scale) extends from (0) where the therapist did not adhere to that aspect of therapy (non-adherence) to (6) where there is adherence and very high skill. Thus the scale assesses both adherence to therapy method and skill of the therapist. To aid with the rating of items of the scale, an outline of the key features of each item is provided at the top of each section. A description of the various rating criteria is given in the right-hand margin – see example below in Figure 1.

The examples are intended to be used as useful guidelines only. They are not meant to be used as prescriptive scoring criteria, rather providing both illustrative anchor points and guides.

**Adjusting the Scale in the Presence of Patient Difficulties**

The scale’s dimensions were devised for patients assessed as being well/moderately suited for cognitive therapy (Safran & Segal, 1990). As such, adjustments may need to be made when patient difficulties are evident (e.g. excessive avoidance). Indeed, with problematic patients it is sometimes difficult to apply CT methods successfully; that is, with desirable change. In such circumstances the rater needs to assess the therapist’s therapeutic skills in the application of the methods. Thus even though the therapist may be unsuccessful at promoting change, credit should be given for demonstrations of appropriate skilful therapy.

Safran, J.D. & Segal, Z.V. (1990) *Interpersonal processes in cognitive therapy*. New York, Basic Books.

**Figure 1: Example of the Scoring Layout**

Key features: this is an operationalised description of the item (see examples within the CTS-R).

Mark with an 'X' on the vertical line, using whole and half numbers, the level to which you think the therapist has fulfilled the core function. The descriptive features on the right are designed to guide your decision.

N.B. When rating, take into consideration the appropriateness of therapeutic interventions for stage of therapy and perceived patient difficulty.

**Competence Level Examples**

|  | 0  *Incompetent*  **{{{{{{** | absence of feature, or highly inappropriate performance |
| --- | --- | --- |
|  | 1  *Novice* | Inappropriate performance, with major problems evident |
| *Advanced beginner* | 2 | evidence of competence, but numerous problems and lack of consistency |
| *Competent* | 3 | competent, but some problems and/or inconsistencies |
| *Proficient* | 4 | good features, but minor problems and/or inconsistencies |
| *Expert* | 5 | very good features, minimal problems and/or inconsistencies |
|  | 6 | excellent performance, or very good even in the face of patient difficulties |

* The present scale has incorporated the Dreyfus system (Dreyfus, 1989) for denoting competence, which is described fully in the manual. Please note that the top marks (i.e. near the ‘expert’ end of the continuum) are reserved for those therapists demonstrating highly effective skills, particularly in the face of difficulties (i.e. highly aggressive or avoidant patients; high levels of emotional discharge from the patients; and various situational factors).

The ‘Key Features’ describe the important features that need to be considered when scoring each item. When rating the item, you must first identify whether some of the features are present. You must then consider whether the therapist should be regarded as competent with the features. If the therapist includes most of the key features and uses them appropriately (i.e. misses few relevant opportunities to use them), the therapist should be rated very highly.

The ‘Examples’ are only guidelines and should not be regarded as absolute rating criteria.

**Scoring Distribution**

It is important to remember that the scoring profile for this scale should approximate to a normal distribution (i.e. mid-point 3), with relatively few therapists scoring at the extremes.

Dreyfus, H. L. (1989). The Dreyfus model of skill acquisition. *In* J. Burke (ed.) *Competency based education and training.* London: Falmer Press.

For each item, ensure that you read the *key features* before assessing the therapist on a scale of 0-6 and record the rating on the line next to the item number. Descriptions are provided for every point on the scale.

If the descriptions for a given item occasionally do not seem to apply to the session you are rating, feel free to disregard them and use the more general scale below:

| **0** | **1** | **2** | **3** | **4** | **5** | **6** |
| --- | --- | --- | --- | --- | --- | --- |
| Poor | Barely Adequate | Mediocre | Satisfactory | Good | Very Good | Excellent |

**Part 1: GENERAL THERAPEUTIC SKILLS**

**1.** **AGENDA**

**Key Features:** *In order to make optimal use of time available in the therapy session, discrete, appropriate and realistic topics need to be identified for the session. The agenda should be set in a collaborative way, reflecting both the specific current issues that the client may have in relation to the workbook module completed, and the overall rationale and goals for therapy.*

1. Therapist did not set agenda.

**2** Therapist set an agenda that was vague or incomplete, but major difficulties evident (e.g. unilaterally set). Poor adherence.

**4** Therapist set an agenda consistent with the workbook module that has just been used. Minor difficulties evident (e.g. no prioritisation or limited patient input) but appropriate features covered (e.g. review of homework). Moderate adherence.

**6**  Therapist worked with the patient to set an agenda consistent with the workbook module that has just been used. Good adherence. Three key aspects are (1) setting agenda, (2) with the patient, (3) in relation to the workbook module completed.

**2. DEALING WITH QUESTIONS/ OBJECTIONS/ PROBLEMS**

**Key Features**: *An important aspect of good therapy is to be able to deal with questions from the patient and objections or problems that the patient may have with any aspect of the therapy or therapeutic relationship. The therapist should elicit any problems or objections and deal with them sensitively and directly. This is similar to but distinct from eliciting feedback on the therapy session as a whole.*

1. Therapist fails to acknowledge questions, dismisses them or makes no attempt to answer them.

**2** Therapist showed some evidence of understanding of the patient’s questions, objections or problems, but did not deal with them clearly and/or appropriately (e.g. some misunderstanding, answers may be unclear, client does not understand the answer, or therapist ‘knocked off track’).

**4** Therapist was sensitive to and understood patient’s questions objections or problems and had some success in dealing with them appropriately.

**6** Therapist understood and was fully sensitive to patient’s questions, objections or problems and dealt with them appropriately, OR, in the face of patient difficulties, the therapist clearly linked questions or problems back to model (if appropriate), and elicited feedback from the client.

**3. CLARITY OF COMMUNICATIONS**

**Key Features:** *An essential aspect of good therapy is clarity of communication. This involves clear use of language at a level that is appropriate to the clients’ ability, avoiding jargon, and generally presenting information in a style that is clear and easily understood.*

**0** Therapist overused jargon and was very muddled in their presentation of information, or used language that was highly inappropriate for the clients’ level of understanding.

**2** Therapist presented information in a generally coherent fashion but was overly technical.

**4** Therapist presented information in a generally clear way. May include attempts to use stories, or examples.

**6** Therapist displayed excellent communication skills and presented information in a clear and well-ordered fashion. Used appropriate stories or examples which were highly effective in illustrating specific points, OR effective communication in the face of severe patient difficulties.

**4. PACING AND EFFICIENT USE OF TIME**

**Key Features:** *The sessions should be well ‘time managed’ in relation to the agenda, and allow smooth progression through start, middle and concluding phases. Work must be paced to suit clients’ needs (e.g. learning speed), and important issues need to be followed. The session should not go over time, without good reason.*

**0** Therapist made no attempt to structure therapy time. Session seemed aimless or over rigid. Client dominated session completely.

**2** Reasonable pacing, but digression or repetitions from the therapist and/or client leads to inefficient use of time. Unbalanced allocation of time, or session overran without good reason.

**4** Balanced allocation of time with discrete start, middle and concluding phases. Minor problems evident (e.g. therapist occasionally let the session become dominated by panic behaviours)

**6** Excellent time management, enabling the agenda to be covered in its entirety. Therapist used time very efficiently by tactfully limiting peripheral and unproductive discussion and by pacing the session as rapidly as was appropriate for the patient OR highly effective in the face of difficulties.

**5. INTERPERSONAL EFFECTIVENESS**

**Key Features:** *The client is put at ease by the therapist’s verbal and non-verbal (e.g. listening skills) behaviour. The client should feel that the core conditions (e.g. warmth, genuineness, empathy and understanding) are present. However, it is important to maintain professional boundaries.*

**0** Therapist had poor interpersonal skills. Their manner and interventions make the client disengage and become distrustful and/or hostile (e.g. therapist seemed hostile, demeaning, or in some other way destructive to the patient).

**2** Therapists style (e.g. intellectualisation) at times impedes their empathic understanding of the patients’ communications. Displayed little confidence.

**4**  Therapist displayed a satisfactory degree of warmth, concern, confidence, genuineness and professionalism. No significant interpersonal problems. The therapist is able to understand explicit and implicit meanings of the client communications.

**6** Excellent interpersonal effectiveness. Therapist displayed optimal levels of warmth, concern, confidence, genuineness and professionalism, appropriate for this particular patient in this session OR highly effective in the face of difficulties.

**Part 2: CONCEPTUALISATION, STRATEGY AND TECHNIQUE**

THIS ITEM IS NOT APPLICABLE FOR SESSION 4, 5 and 6

**6. REVIEW OF PANIC DIARIES**

**Key Feature:** *Self-monitoring is an important part of therapy, which the client should be actively engaged in between sessions. In order to monitor changes (which are often minimised by clients once they have managed to achieve a particular goal) it is important that these activities are monitored and measured. The panic diary is important to identify particular patterns of symptoms, triggers, and situations. The diary is also used to identify beliefs and safety-seeking behaviours which can be linked to the cognitive model.*

1. Therapist did not look at the panic diary.

**2** Therapist looked at the diary but commented on it in a cursory and unhelpful fashion.

**4** Therapist reviewed diary, noted any changes in panic frequency and had some success in capitalising on these changes. Commented on these changes in the diary in a helpful way.

**6** Therapist reviewed diary in a collaborative manner, noted any increase or decrease in panic frequency, elicited patient’s explanation for such changes. Therapist linked the events to the model and commented in a full and helpful way.

**7. REVIEWING WORKBOOK MODULES**

**Key Features:** *The workbook modules are an essential part of focused CBT in order to aid learning, test out ideas, develop new understanding, and try out new experiences. In order to ascertain how successful the client has been with the workbooks, and what they have learned, it is important to allocate time to review the workbooks and use the information to lead into the session and direct future homework.*

1. Therapist did not review previous workbook.

**2** Therapist reviewed workbook, noted outcome, but did not attempt to elicit what the client had learned from the experience. Some evidence of competence, but numerous problems evident.

**4** Therapist reviewed previous workbook and had some success in clarifying its outcome or what the patient had learned from the workbook. Minor problems evident (e.g. insufficient time allowed for discussion) but generally competent.

**6** Therapist skilfully reviewed previous workbook, identified any problems, established the outcome of the workbook assignment and worked with the patient to maximise what could be learned from the assignment and developed in the session. The therapist identified how any new learning could be integrated into daily life OR therapist performance was highly effective in the face of difficulties.

**8. USE OF FEEDBACK AND SUMMARIES**

**Key Features:** *The clients and therapists understanding of key issues should be helped through the use of two-way feedback. The main ways of doing this are through the use of a general summary and chunking important units of information (capsule summaries). The therapist should seek regular feedback from the client to help him/her understand the patients’ situation and to ascertain the clients understanding of therapy and to facilitate the client’s ability to gain new insights and make therapeutic shifts. It also keeps the patient focused.*

**0** Therapist did not ask for feedback to determine patients understanding of things covered during the session or provide summaries.

**2** Therapist elicited some feedback from patient but did not ask enough questions to be sure that the patient understood the line of reasoning or was satisfied with the session. Appropriate feedback not given frequently enough by therapist, with insufficient attempts to elicit and give feedback (e.g. feedback too vague to provide opportunities for understanding and change).

**4** Therapist asked sufficient questions to be sure that the patient understood the line of reasoning and to determine their response to the session. Therapist adjusted their behaviour in response to feedback, when appropriate. Appropriate feedback given and elicited during the session, facilitating therapeutic gains. Session summarised at the end. Minor problems evident (e.g. inconsistent, or didactic).

**6** Therapist was especially adept at eliciting and responding to verbal and non-verbal feedback throughout the session (e.g. regularly checked for understanding during session, asked patient to summarise at the end of the session and helped fill in any gaps in the summary). Time for therapist and patient to reflect on the session OR therapist performance was highly effective in the face of difficulties.

**9. GUIDED DISCOVERY**

**Key features:** *The client should be helped to explore their difficulties and then develop hypotheses regarding their current situation and to generate potential solutions for themselves through guided discovery. To facilitate this, the therapist should maintain an open and inquisitive style and use Socratic questioning to lead the client towards new ways of looking at things. Did the therapist use didactic persuasion to urge the client to change their beliefs? Did the therapist work with the client to determine what the realistic consequences would be if the client’s beliefs proved to be true? Did the therapist help the client to view the available evidence or information (including the client’s prior experiences) to test the validity of the client’s beliefs?*

**0**  No attempt at guided discovery. Therapist seemed to be “cross-examining” patient, putting the patient on the defensive, or forcing his/her point of view on the patient.

**2** Minimal opportunity for discovery. Some use of a questioning style which is following a productive line of discovery.

**4** Therapist, for the most part, helped the patient explore and then see new perspectives through guided discovery (e.g. examining evidence, considering alternatives) rather than through debate. Used questioning appropriately. Minor problems evident (e.g. some inconsistency, occasionally lapsing into over long didactic approach). Used other methods e.g. teaching about beliefs as appropriate.

**6** Therapist was especially adept at using guided discovery during the session to explore panic related problems and help patient draw their own conclusions. Achieved an excellent balance between skilful questioning and other modes of intervention. Able to integrate significant client interruptions or difficulties OR therapist performance was highly effective in the face of difficulties.

**10. FOCUS ON PANIC RELATED COGNITIONS, APPROACH SUPPORTING AND SAFETY SEEKING BEHAVIOURS**

**Key Features:** *The main feature of focused CBT for panic disorder is the focus on panic related cognitions, and the way that they generate bodily sensations and anxious feelings. Specifying how panic related cognitions are maintained by safety seeking behaviours and other strategies, is an essential feature of treatment. Using approach supporting behaviours is also an important part of treatment. The therapist should ask the client to report specific thoughts that the client experienced either in the session or in a situation that occurred prior to the session. Thoughts should be linked to the vicious circle model and linked to specific safety seeking behaviours that are intended to prevent the feared catastrophe. At the appropriate stage of therapy the therapist explores with the client a general belief that underlies many of the client’s specific negative/feared thoughts and beliefs. The therapist probes beliefs and safety seeking behaviours and regularly links them back to the vicious circle model. The therapist collaboratively identifies appropriate approach supporting behaviours the patient can use.*

**0** Therapist failed to elicit misinterpretations of bodily sensations or to discuss their role in panic attacks. Not accessing cognitions.

**2** Therapist discussed in general terms the role of misinterpretations of bodily sensations and safety seeking behaviours play in the production of panic. However, the therapist failed to focus on the patient’s specific misinterpretations, specific safety seeking behaviours or the link between the two and may not have identified approach supporting behaviours they can use.

**4** Therapist elicited and discussed specific misinterpretations of bodily sensations and established their role in the production of panic attacks. However, the therapist failed to obtain belief ratings. Therapist identified the safety seeking behaviours used by the patient to prevent catastrophe, but some problems evident with the disconfirmatory role of the safety seeking behaviours. Therapist and patient collaboratively identified approach supporting behaviour(s) the patient can use but problems with differentiating them with safety seeking behaviours/implementation of them.

**6** Therapist skilfully elicited and discussed specific misinterpretations of bodily sensations, and established their role in the production of panic attacks. Therapist took ratings, and established specific links between safety seeking behaviours and cognitions and monitored changes in belief. Therapist and patient collaboratively identified approach supporting behaviours that can be used and was able to differentiate them with safety-seeking behaviours. Alternatively, the therapist’s performance was highly effective in the face of difficulties.

**11. RATIONALE**

**Key Features:** *The format of therapy is clearly explained. Procedures closely follow an explicit theoretical model which is made clear by the therapist and understood by the client. The therapist provided a rationale which emphasised the importance of evaluating the accuracy of the client’s beliefs and changing inaccurate beliefs. The therapist used a mini rationale to explain to the reasons for pursuing a particular topic or intervention in the session.*

**0** Therapist used procedures without adequate and explicit rationale. No attempt to establish clients understanding of procedures used.

**2** Therapist tended to give incomplete and/or unclear rationale for procedures used. Some attempt to check clients understanding, but explicit feedback not sought and misunderstandings not clearly identified or addressed.

**4** Therapist gave complete and clear rationale. Clients understanding is established, feedback sought and difficulties/misunderstandings addressed. Minor difficulties evident (e.g. unclear at times, explanations not always complete).

**6** Excellent rationale given clearly, clients understanding checked, feedback sought, and misunderstandings addressed collaboratively or appropriate rationale delivered clearly, OR therapist performance was highly effective in the face of difficulties.

THIS ITEM IS NOT APPLICABLE UNTIL BEHAVIOURAL EXPERIMENTS ARE USED IN TREATMENT. PRO-RATA SCORE IF N/A

**12. SELECTION OF APPROPRIATE STRATEGIES FOR COGNITIVE CHANGE INCLUDING SELECTION OF BEHAVIOURAL EXPERIMENTS IN RELATION TO THE WORKBOOK MODULE COMPLETED.**

***Key Features:*** A range of techniques can be used to promote cognitive change. These include identifying alternative explanations of bodily sensations, reviewing the evidence for the patient’s catastrophic interpretations, challenging relevant assumptions or beliefs, designing behavioural experiments etc. Normally a mixture of techniques for cognitive change would be used (verbal, imagery, behavioural experiments). Note: focus on the quality of the therapist’s strategy, not on how effectively the strategy was implemented. The best selection of techniques will be those aimed at key beliefs. The strategies chosen should be in line with the workbook module completed.

1. Therapist did not select techniques for cognitive change. Therapist did not design any appropriate behavioural experiments.

**2** Therapist selected specific techniques/experiments for cognitive change. However, the overall therapeutic strategy seemed vague or the techniques did not seem promising for this patient or the strategy chosen was not in line with the workbook module completed.

1. Therapist seemed to have a generally coherent therapeutic strategy, which showed reasonable promise and incorporated appropriate techniques for cognitive change. Therapist seemed to have a generally coherent behavioural experiment, and the client understood the rationale. The experiment included some aspects of predictions and safety seeking behaviours/approach supporting behaviours (if appropriate) and showed reasonable promise for cognitive change.

**6** Therapist followed a coherent, consistent therapeutic strategy, which seemed very promising and incorporated the most appropriate techniques for cognitive change. Therapist set up a coherent experiment that had a clear rationale, understood by the client, and predictions with ratings. They seemed very promising for cognitive change. Specific and relevant safety seeking and approach supporting behaviours were identified, rated, and their use addressed OR therapist performance was highly effective in the face of difficulties.

THIS ITEM IS NOT APPLICABLE UNTIL BEHAVIOURAL EXPERIMENTS ARE USED IN TREATMENT. PRO-RATA SCORE IF N/A

**13. APPROPRIATE IMPLEMENTATION OF TECHNIQUES FOR COGNITIVE CHANGE AND IMPLEMENTATION OF BEHAVIOURAL EXPERIMENTS IN RELATION TO THE WORKBOOK MODULE COMPLETED.**

**Key Features:** *A range of techniques can be used to promote cognitive change. These include identifying alternative explanations of bodily sensations, reviewing the evidence for the patient’s catastrophic interpretations, challenging relevant assumptions or beliefs, changing panic-related images, designing behavioural experiments etc. Note: focus on the how effectively the strategy was implemented or adapted and ensure the strategy implemented was in line with the workbook module completed.*

**0** Therapist did not apply any appropriate techniques. Therapist did not implement any behavioural experiments. Therapist misuses techniques.

**2** Therapist used appropriate techniques, but there were major flaws in the way they were applied. Therapist used appropriate experiments, but there were major flaws in the way they were conducted. For example, therapist did not take specific key predictions or check that safety seeking behaviours were dropped. Or, the implemented technique was not in line with the workbook module completed.

1. Therapist applied appropriate techniques with moderate skill. Therapist used specific experiments with moderate skill, in line with the workbook modules completed. The therapist “got out of their chair”, and modelled first in experiments/completed the experiments with the patient, where appropriate. Therapist took predictions and checked safety seeking behaviours were dropped. Applies a range of methods with skill and flexibility, enabling client to develop new perspectives. Minor problems evident.

**6** Therapist very skilfully and resourcefully applied a range of appropriate techniques. Therapist very skilfully and resourcefully used specific experiments targeted at key specific beliefs, both in-session and set as homework and were in line with the workbook modules. Specific key predictions were made and rated pre- and post- experiment. Results were discussed and taken forward to homework, next steps, or new experiments. Therapists successfully adapted methods in the face of significant client difficulties or challenges.

THIS ITEM IS NOT APPLICABLE UNTIL BEHAVIOURAL EXPERIMENTS ARE USED IN TREATMENT. PRO-RATA SCORE IF N/A

**14. INTEGRATION OF DISCUSSION AND BEHAVIOURAL TECHNIQUES**

**Key Features:** *Cognitive therapy for panic disorder requires integration of both verbal and imagery techniques, such as examining evidence for beliefs and behavioural experiments to test predications made by panic related beliefs. This item focuses on the manner in which the therapist uses and moves between strategies. Whilst related to ‘selection and implementation of techniques for cognitive change and behavioural experiments’, this item is more concerned with how one flows into the other. Ideally, discussion techniques should naturally lead into establishing behavioural experiments in order to discover new information and evidence.*

**0** No discussion of evidence for beliefs or behavioural experiments.

**2** Therapist used both discussion and behavioural experiments, but not linked together.

**4** There was good flow between discussion and behavioural techniques. They were linked together and gathered new evidence to explore and test the beliefs. Both discussion and behavioural techniques were used to examine the same belief.

**6** There was seamless flow between discussion and behavioural experiments. Both were done very well and linked very clearly to the specific beliefs OR therapist performance was highly effective in the face of difficulties.

THIS ITEM IS NOT APPLICABLE AT THE FINAL SESSION

**15. HOMEWORK SETTING**

**Key Features:** *Homework tasks should be appropriate for the stage of therapy, consistent with the conceptualisation, and have precise and clear goals. Homework should always be reviewed at the next session and should be in line with the next workbook module.*

**0** Therapist did not set relevant homework.

**2** Therapist set somewhat inappropriate, vague or over-general homework. Therapist negotiates homework unilaterally and in a routine fashion, without explaining the rationale for new homework. Homework set was not in line with the next workbook module.

**4** Appropriate new homework jointly negotiated with clear goals and rationales and in line with the next workbook module. Homework linked not only to general issues but to specific cognitions or behaviours identified in the session. However, minor problems evident.

**6** Excellent homework negotiated including the testing or exploring of key cognitions, OR highly appropriate homework set in the face of difficulties.

**Results**:

| Total score on all 15 items (max. possible score is 90) |  |
| --- | --- |
| Percentage score |  |

**OVERALL RATINGS AND COMMENTS**

**Key Features:**  *Particular criterion need to be met for the standard of therapy to meet the requirement of treatment trials. The rating is not a question of ‘is this a good therapist?’, or whether the intervention was good or effective, but whether the method and standard of delivery is consistent with the stringent requirements of a treatment trial. As such, it is important to strive for homogeneity amongst trial therapists. The nature of the presenting problem is not necessarily the defining feature in terms of client difficulty, but involves factors such as interpersonal style, level of comprehension, ‘psychological mindedness’ etc.*

1. How well did the clinician adhere to the session guide?

|  |  |  |  |  |  |  |
| --- | --- | --- | --- | --- | --- | --- |
| **0** | **1** | **2** | **3** | **4** | **5** | **6** |
| **Not at all** | **Below substandard** | **Substandard** | **Satisfactory** | **Good** | **Very Good** | **Excellent** |

1. How would you rate the clinician overall in this session as a therapist using low intensity cognitive therapy for panic? (Ratings of 4 and above would indicate suitability as a therapist on a controlled trial for CT in panic).

|  |  |  |  |  |  |  |
| --- | --- | --- | --- | --- | --- | --- |
| **0** | **1** | **2** | **3** | **4** | **5** | **6** |
| **Poor** | **Barely** | **Mediocre** | **Satisfactory** | **Good** | **Very Good** | **Excellent** |

1. How difficult did you feel this patient was to work with?

|  |  |  |  |  |  |  |
| --- | --- | --- | --- | --- | --- | --- |
| **0** | **1** | **2** | **3** | **4** | **5** | **6** |
| **Not Difficult** |  |  | **Moderately Difficult** |  |  | **Extremely Difficult** |

REASONS/COMMENT:

Appendix G – Panic Safety-Seeking and Approach Supporting Behaviours Questionnaire

When you are at your most anxious or panicky, how often do you do the following things as a way of protecting yourself against harm?

Please highlight or circle your answers.

| Try to think about other things | Never | Sometimes | Often | Always |
| --- | --- | --- | --- | --- |
| Hold on to or lean on to something | Always | Often | Sometimes | Never |
| Hold on to or lean on someone | Never | Sometimes | Often | Always |
| Sit down | Always | Often | Sometimes | Never |
| Keep still | Always | Often | Sometimes | Never |
| Move very slowly | Never | Sometimes | Often | Always |
| Look for an escape route | Never | Sometimes | Often | Always |
| Make yourself do more physical exercise | Always | Often | Sometimes | Never |
| Focus attention on your body | Always | Often | Sometimes | Never |
| Try to keep control of your mind | Never | Sometimes | Often | Always |
| Try to keep tight control over behaviour | Always | Often | Sometimes | Never |
| Talk more | Never | Sometimes | Often | Always |
| Take medication | Never | Sometimes | Often | Always |
| Ask people around for help | Never | Sometimes | Often | Always |
| Change your breathing | Always | Often | Sometimes | Never |

Panic Approach Supporting Behaviours Questionnaire

When you are doing things to overcome your anxiety and panicky feelings, how often do you do the following things to help you confront the fearful situations more effectively?

| Focus on things which are important to me | Never | Sometimes | Often | Always |
| --- | --- | --- | --- | --- |
| Be decisive in what I do | Always | Often | Sometimes | Never |
| Take rests before moving on | Never | Sometimes | Often | Always |
| Talk to people around me | Always | Often | Sometimes | Never |
| Remind myself of the benefits of confronting my anxiety | Always | Often | Sometimes | Never |
| Focus on my surroundings and what’s around me | Never | Sometimes | Often | Always |
| Tell myself that if I stick it out my anxiety and panic will diminish | Never | Sometimes | Often | Always |
